# Self-harm in children involved in private and public family justice court proceedings: longitudinal national data linkage study

**DOI:** 10.1192/bjo.2025.10971

**Published:** 2026-02-27

**Authors:** Ann John, Joanna McGregor, Lucy J. Griffiths, Rhodri Johnson, Karen Broadhurst, Amanda Marchant

**Affiliations:** Population Data Science, Swansea University Medical School, Swansea, UK; Centre for Child & Family Justice Research, Lancaster University, Lancaster, UK; National Centre for Suicide Prevention and Self-Harm Research, Swansea University, Swansea, UK

**Keywords:** Care proceedings, administrative data, data linkage, children, self-harm

## Abstract

**Background:**

Little is known about self-harm in children involved in family justice proceedings, particularly in private family courts in England and Wales.

**Aims:**

To examine records of self-harm in children involved in private and public law proceedings using population-level linked data.

**Method:**

A retrospective e-cohort study of children aged under 18 years, using linked health and family justice (Cafcass Cymru) data (2011–2018). Family court involvement was recorded from age 0 to 17 years. Incidence of self-harm was recorded from age 10 to 17 years to fit with the standard definition of self-harm. Annual incidence of self-harm over time across general practitioner (GP), emergency department and hospital admissions for individual children in private and public law proceedings were compared with a non-court cohort using Poisson regression. Self-harm following court proceedings was compared with an age- and gender-matched non-court cohort using Cox regression.

**Results:**

Adjusted self-harm rates were higher in court-involved children than the non-court cohort (incident rate ratios (IRRs) (95% CI), private: GP 1.8 (1.6–2.1); emergency department 1.4 (1.2–1.7); admissions 1.8 (1.5–2.1); public: GP 4.6 (4.1–5.3); emergency department 5.0 (4.3–5.8); admissions 5.0 (4.3–5.8)). Compared with matched comparison children, risk of self-harm was higher following private (adjusted hazard ratios 2.0 (1.7–2.2)) and public court proceedings (hazard ratio 2.3 (2.7–3.8)). Hazard ratios were greater for those from less deprived areas and those with no history of self-harm.

**Conclusions:**

The elevated risk of self-harm in children involved in public law proceedings is well recognised. Our study highlights risk in children in private family justice proceedings. Elevated risk among those from less deprived areas and those with no history of self-harm may reflect circumstances associated with family justice involvement, resulting in rates comparable to children with other pre-existing vulnerabilities. Contact with family justice is an opportunity to offer preventative support.

The safeguarding and welfare interests of children involved in family justice proceedings in England and Wales are represented by a government organisation ‘Children and Family Court Advisory and Support Service’ (Cafcass (https://www.cafcass.gov.uk/), Cafcass Cymru in Wales (https://www.gov.wales/cafcass-cymru)). Decisions on caregiver arrangements, family contact and the living arrangements of the children involved are made in private and public family court proceedings. Private law cases are brought to court by individuals, generally in connection with divorce or parental separation. An estimated 10% of separated families resort to this.^1^ Public law cases are instigated by local authorities and relate to the safety of children, and can lead to a child being taken into care, adopted, fostered or special guardianship and supervision orders being made. Across England and Wales, 51 658 private and 19 037 public law cases were initiated in 2018.^2^ Around a third of private court proceedings in Cafcass Cymru data have a marker of welfare concerns.^3^ Children involved in family law proceedings may have been exposed to a range of adverse childhood experiences (ACEs) including poverty, homelessness, domestic abuse, household member mental health difficulties or substance abuse, as well as parental separation.^4^

Self-harm refers to any act of intentional self-injury or self-poisoning regardless of suicidal intent or motivation.^5^ Rates of self-harm in children and young people (CYP) have increased in recent years in the UK,^6^ particularly in adolescent females.^7^ Self-harm is one of the strongest risk factors for future suicide.^8^ Recent Office of National Statistics statistics reported the largest increase in suicide rates in females aged under 24 since recording began in 1981.^9^ Parental divorce, childhood maltreatment, household stress, financial stress and poor family relationships are risk factors for self-harm in young people.^10^ Previous research with routinely collected data found that looked-after-children are at a higher risk of self-harm and suicide compared with non-care populations.^11,12^ However, little is known about rates of self-harm for the wider population of children in contact with family courts. Previous research using Cafcass Cymru data has demonstrated that children involved in both private and public family court proceedings are at an increased risk of depression and anxiety.^13^ Accounts from parents describe self-harm and suicidal ideation in children following private family justice proceedings.^14^ However, an absence of quantitative data and comparisons with the general population were found in an evidence review.^15^ To our knowledge, no study has yet quantified self-harm in this population or compared rates of self-harm to the general population.

## Aims and objectives

This study aimed to assess self-harm in children involved in family court proceedings compared with children with no court experience. We examined rates of self-harm and trends over time across primary and secondary healthcare settings and self-harm as an outcome following court proceedings. This was explored for children involved in private and public family court proceedings and a non-court comparison cohort to provide evidence for policy making, service planning and resource allocation.

## Method

### Study design

This was a retrospective e-cohort study. A matched cohort design was used to investigate risk of self-harm following initiation of court proceedings. Results are reported according to the Strengthening the Reporting of Observational Studies in Epidemiology checklist.

### Data source

The Adolescent Mental Health Data Platform (ADP; www.adolescentmentalhealth.uk) is a data platform that supports mental health research in CYP through research-ready data, code lists and algorithms to enhance mental health data science specifically for CYP. For this study, the ADP utilised data-sets held within the Secure Anonymised Information Linkage (SAIL) Databank, an expanding ISO 27001 certified data repository of person-based-data linkable across healthcare and public settings to support research. Policies, control measures and practices have been described previously.^16,17^ All data were treated in accordance with the Data Protection Act 2018.

### Study population and setting

The following data-sets were linked at individual level: the Welsh Demographic Service (WDS); the Welsh Index of Multiple Deprivation (WIMD) containing deprivation scores for all lower super output areas in Wales; the Welsh Longitudinal General Practice data-set (WLGP) at the time of analysis, approximately 80% of all general practices (GPs) in Wales were supplying SAIL with their data; the Emergency Department Dataset (EDDS), which contains National Health Service (NHS) Wales emergency department attendances (34 sites, including minor injury units); the Patient Episode Database for Wales (PEDW) containing data for all NHS Wales hospital admissions; Cafcass Cymru containing a routinely produced extract of administrative case management.^18^ At time of study, the SAIL Databank held information for both private and public law cases conducted between 1 January 2011 and 31 December 2018. All data-sets were linked at individual level using an anonymous linkage field (ALF). Of the children involved in private and public samples during the study period, 87% were assigned an ALF enabling linkage of their information to the other data sources within SAIL.^16,18^

Data were collected between the 1 January 2011 and 31 December 2018. Individuals aged under 18, registered to a SAIL-supplying GP during this period, with at least 12 months’ worth of continuous GP data, were included in the study.

Data collection began from six months post-GP-registration or study onset, whichever was the latest and it ended on the date of GP de-registration, the child’s 18th birthday, death or the end of the study period, whichever was the earliest.

We identified children involved in private and public family law proceedings from birth to 17 years of age. Children who had been involved in both private and public family court proceedings were assigned to the public court proceedings cohort to avoid loss of this data. For the children involved in court proceedings on more than one occasion, we used the first court registration date within a period of registration to a SAIL-supplying GP.

We identified our non-court comparison cohort from the WDS data-set. These were children of the same age registered with a SAIL-supplying GP over the study period (meeting all other criteria above) who did not have a record of involvement with family court proceedings.

### Measures

Self-harm events were identified in GP, emergency department and hospital admissions data-sets in individuals aged 10–17. All codes and methodologies implemented have been published previously.^19,20^ An incident self-harm event in this study was characterised as a record of self-harm, with no episode recorded within the previous 12 months.

Demographic information was collected from the WDS data-set. Age and deprivation information for each individual was collected based on the start of data collection for each year for the incidence measures and on the date of the first court application for the time-to-event analyses (described below). Age was divided into bands (10–14, 15–17 years). Data on gender assigned at birth was collected (gender identity not available). The date and type of court application submitted was collected from Cafcass Cymru data.

### Statistical analyses

#### Descriptive statistics

We describe the demographics (number, gender, age range, median, interquartile range and deprivation) of the private, public and non-court cohorts with counts and proportions of individuals who have had a previous history of self-harm. All confidence intervals for proportions were estimated by the Wilson score method with continuity correction.^21^

#### Incidence measures

We calculated annual incidence rates (using person years at risk (PYAR) as the denominator. Poisson regression was used to calculate incidence rate ratios (IRRs) of self-harm, adjusting for age, gender, deprivation and year for the private, public and non-court cohorts plus a further model that included all cases with court itself (private, public or non-court) as a covariate. The amount of dispersion in the model was determined by the Pearson chi-square goodness-of-fit statistic. Where there was evidence of over-dispersion, the analysis was run using negative binomial regression. The significance of the variables was assessed by Wald tests. All confidence intervals for rates were estimated using two-tailed mid-*p* exact confidence intervals.^22^

#### Time-to-event analyses

Time-to-event analyses were conducted to assess the impact of involvement on court proceedings on the risk of self-harm. We identified ten controls per case matched on age and gender with SAIL-supplying GP data. We modelled the length of time from the first recorded date of registration with the court to the first record of self-harm, or to censorship (i.e. end of follow-up).

We performed conditional (pairwise) Cox regression to derive unadjusted and adjusted hazard ratios with 95% confidence intervals, comparing risk of self-harm event following exposure to court to those with no court involvement. The proportional hazards assumption was tested graphically using Kaplan–Meier and log-minus-log curves. We fitted univariate and multivariate models, adjusting for deprivation at court date and history of self-harm, and a further model with court type (private, public) as a covariate itself. Survival curves, with significance of difference assessed by log-rank tests, were plotted.

SQL (DB2) and R (version 4.1.3 in R Studio version 03.10.2022) were used to query the database and extract the data. Statistical analyses were conducted using SPSS statistical software (version 26). All analyses were completed in Windows 10.

### Ethical approval and consent to participate

The ethical considerations of this project were covered by permissions granted to the MytHICAL (Mental Health Informatics in Children, Adolescents and young adults) programme. Additional approval (SAIL Project No: 1040) was granted on 23 April 2020 by the Swansea University Information Governance Review Panel, an independent body consisting of government, regulatory and professional agencies that grants approval for projects conducted within the SAIL Databank. Cafcass Cymru (the data owner of the family courts data) also approved use of the data for this project.

### Patient and population involvement

This study was guided by advocates for children throughout, including heads of children’s services across Wales. It was discussed with the Wolfson Young Persons Advisory Group as part of wider work in relation to children in public court proceedings and care experience.

### Role of the funding source

The funders did not have any role in study design, collection, analysis and interpretation of data, in the writing of the report or the decision to submit for publication.

## Results

### Study population

The study population comprised 703 182 children (aged under 18), registered to a SAIL-supplying GP between 1 January 2011 and 31 December 2018 (Table 1). Records of family court involvement are reported for those aged 0–17 and records of self-harm are reported for those aged 10–17 years.

**Table 1:** Demographic breakdown of cohorts at study onset

Variable	Non-court (*n*)	Proportion (% (95% CI))	Private (*n*)	Proportion (% (95% CI))	Public^a^ (*n*)	Proportion (% (95% CI))
Total	680 617		17 041		5524	
Gender						
Male	348 647	51.2 (51.1–51.4)	8744	51.3 (50.6–52.1)	2840	51.4 (50.1–52.7)
Female	331 970	48.8 (48.7–48.9)	8297	48.7 (47.9–49.4)	2684	48.6 (47.3–49.9)
Age group						
<10	461 182	67.8 (67.7–67.9)	15 702	92.1 (91.7–92.5)	4888	88.5 (87.6–89.3)
Ages 10–14	154 013	22.6 (22.5–22.7)	1297	7.6 (7.2–8.0)	600	10.9 (10.1–11.7)
Ages 15–17	65 422	9.6 (9.5–9.7)	42	0.2 (0.2–0.3)	36	0.7 (0.5–0.9)
Deprivation^b^						
Least deprived	122 365	18.0 (17.9–18.1)	2142	12.6 (12.1–13.1)	274	5.0 (4.4–5.6)
Second least deprived	108 270	15.9 (15.8–16.0)	2311	13.6 (13.1–14.1)	440	8.0 (7.3–8.7)
Middle deprived	127 366	18.7 (18.6–18.8)	3018	17.7 (17.1–18.3)	785	14.2 (13.3–15.2)
Second most deprived	139 674	20.5 (20.4–20.6)	3984	23.4 (22.8–24.0)	1283	23.2 (22.1–24.4)
Most deprived	164 803	24.2 (24.1–24.3)	5272	30.9 (30.3–31.6)	2645	47.9 (46.6–49.2)

a. Includes children and young people involved in both public and private court proceedings (*n* = 563; 2.5% (1.4–2.6)).

b. Deprivation data not available for all individuals.

**Fig. 1:**
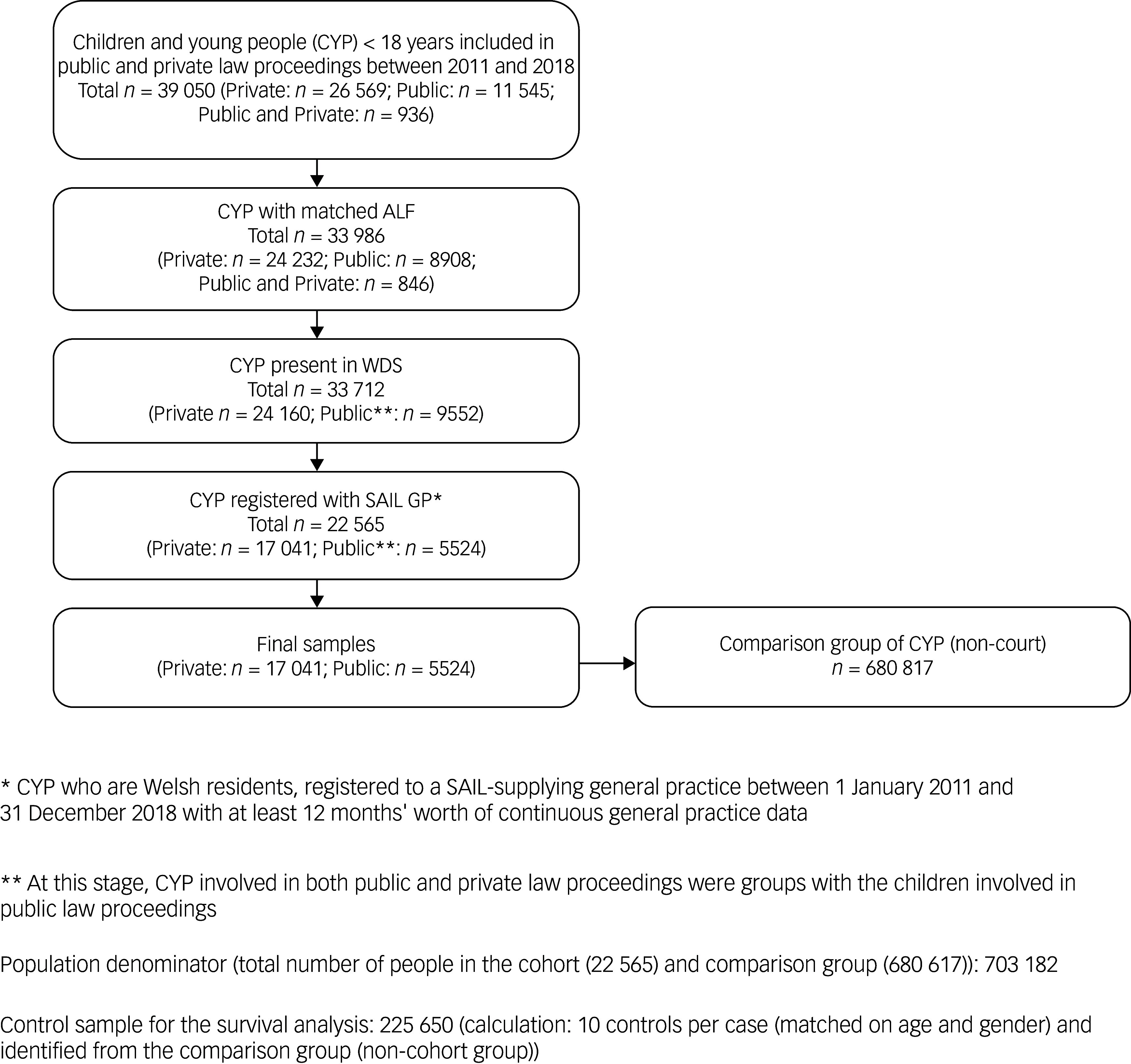
Flow diagram of study participants. ALF, anonymous linkage field; WDS, Welsh Demographic Service; SAIL, Secure Anonymised Information Linkage; GP, general practitioner.

The final sample consisted of 17 041 children involved in private law proceedings only, and 5524 children involved in public law proceedings (includes those registered in both private/public) whose records could be linked to routinely collected healthcare data. We identified 680 617 children without a family court record (non-court cohort; Fig. 1). Out of the cohorts, the greatest proportion of children living in the most deprived quintile were within the ‘public court’ cohort (47.9% (46.6–49.2)), followed by ‘private court’ (30.9% (30.3–31.6)), and finally the ‘non-court’ cohort (24.2% (24.1–24.3)).

### Incidence of self-harm

Adjusted IRRs of incidence of self-harm by healthcare setting and private/public court proceedings are shown in Table 2. With the ‘non-court’ cohort as a reference, incidence of self-harm was around twice as high for the ‘private court’ group (IRRs: GP 1.8 (1.6–2.1), emergency department 1.4 (1.2–1.7), hospital admissions 1.8 (1.5–2.1)) and more than four times as high in the ‘public court’ group (IRRs: GP 4.6 (4.1–5.3), emergency department 5.0 (4.3–5.8), hospital admissions 5.0 (4.3–5.7)).

**Table 2:** Self-harm incidence rates, IRR (unadjusted, adjusted and 95% CI) across healthcare settings

Setting/Group	*n*	Incidence rates	IRR (adj^a^)	IRR (unadj)
General practitioner				
Non-court	7315	4.4 (4.3–4.5)	Reference (*p* < 0.001)	Reference (*p* < 0.001)
Private	216	7.1 (6.2–8.1)	1.8 (1.6–2.1)	1.6 (1.3–2.0)
Public	213	19.6 (17.0–22.4)	4.6 (4.1–5.3)	4.4 (3.6–5.5)
Emergency department				
Non-court	4524	2.7 (2.7–2.8)	Reference (*p* < 0.001)	Reference (*p* < 0.001)
Private	132	4.3 (3.6–5.1)	1.4 (1.2–1.7)	1.2 (0.9–1.5)
Public	145	13.3 (11.3–15.7)	5.0 (4.3–5.8)	4.7 (3.7–5.8)
Hospital admissions				
Non-court	4881	3.0 (2.9–3.0)	Reference (*p* < 0.001)	Reference (*p* < 0.001)
Private	107	3.5 (2.9–4.2)	1.8 (1.5–2.1)	1.6 (1.2–2.0)
Public	150	13.8 (11.7–16.2)	5.0 (4.3–5.7)	4.9 (3.9–6.1)

IRRs, incident rate ratios; adj, adjusted; unadj, unadjusted.

a. Adjusted IRRs adjusted for year, age, gender and deprivation.

Incidence per 1000 PYAR and IRRs of self-harm by court group, demographic variables and setting are shown in Tables 3 and 4. Incidence is higher (two to four times) in females than in males across all settings. This gender difference was smallest for emergency department attendances and highest for hospital admissions across all groups. Across all settings, rates of self-harm were two to four times higher in 15- to 17-year-olds than 10- to 14-year-olds.

**Table 3:** Incidence rate (95% CI) per 1000 person years at risk for self-harm by court involvement, healthcare setting, year, gender, age group and deprivation

	Non-court			Private court			Public court		
Variable	GP	ED	Hospital admissions	GP	ED	Hospital admissions	GP	ED	Hospital admissions
Year									
2011	3.4 (3.2–3.7)	1.9 (1.7–2.1)	2.0 (1.8–2.2)	3.6 (1.2–8.3)	0.7 (0.0–4.0)	1.4 (0.2–5.1)	14.1 (6.5–26.8)	4.7 (1.0–13.8)	7.8 (2.5–18.3)
2012	4.3 (4.0–4.6)	2.7 (2.5–2.9)	2.5 (2.3–2.7)	5.7 (2.8–10.1)	2.6 (0.8–6.0)	3.6 (1.4–7.4)	19.9 (11.6–31.8)	9.3 (4.0–18.4)	7.0 (2.6–15.3)
2013	5.2 (4.9–5.5)	3.1 (2.9–3.4)	3.1 (2.9–3.3)	7.7 (4.7–11.9)	3.8 (1.8–7.1)	5.0 (2.7–8.6)	21.2 (13.4–31.8)	7.4 (3.2–14.5)	12.9 (7.1–21.7)
2014	4.6 (4.3–4.9)	2.9 (2.7–3.2)	2.9 (2.7–3.1)	6.1 (3.7–9.4)	1.2 (0.3–3.1)	3.3 (1.7–6.0)	16.1 (10.0–24.7)	16.1 (10.0–24.7)	17.7 (11.2–26.5)
2015	4.7 (4.4–5.0)	3.7 (3.4–4.0)	3.3 (3.1–3.6)	8.3 (5.7–11.6)	4.9 (3.0–7.5)	5.6 (3.5–8.4)	21.3 (14.6–30.1)	18.6 (12.4–26.9)	12.6 (7.6–19.8)
2016	4.2 (3.9–4.5)	3.1 (2.9–3.4)	2.7 (2.4–2.9)	7.9 (5.6–10.8)	3.2 (1.9–5.3)	5.1 (3.3–7.5)	22.7 (16.2–31.1)	16.9 (11.3–24.3)	13.4 (8.5–20.1)
2017	4.5 (4.2–4.8)	3.0 (2.8–3.2)	2.9 (2.7–3.1)	6.4 (4.5–8.9)	3.7 (2.3–5.6)	4.4 (2.8–6.4)	23.3 (16.9–31.4)	15.7 (10.5–22.6)	17.9 (12.3–25.1)
2018	4.5 (4.2–4.8)	3.2 (2.9–3.4)	2.6 (2.4–2.8)	7.8 (5.8–10.3)	4.7 (3.2–6.7)	4.1 (2.6–5.9)	15.0 (10.1–21.6)	12.5 (8.0–18.5)	11.4 (7.2–17.3)
Gender									
Male	1.8 (1.7–1.9)	1.9 (1.8–2.0)	1.0 (0.9–1.0)	3.6 (2.8–4.7)	2.1 (1.5–3.0)	1.5 (0.9–2.2)	7.7 (5.5–10.4)	7.7 (5.5–10.4)	4.9 (3.2–7.1)
Female	7.2 (7.0–7.4)	4.1 (4.0–4.3)	4.6 (4.5–4.8)	10.7 (9.1–12.5)	5.0 (3.9–6.3)	7.4 (6.0–8.9)	31.2 (26.7–36.2)	19.8 (16.2–23.8)	21.6 (17.9–25.8)
Age group									
10–14 years	3.1 (3.0–3.2)	1.7 (1.6–1.7)	2.0 (1.9–2.1)	5.6 (4.7–6.6)	2.4 (1.8–3.1)	3.2 (2.6–4.0)	16.4 (13.9–19.2)	10.5 (8.5–12.9)	11.5 (9.4–13.9)
15–17 years	7.2 (7.0–7.4)	5.7 (5.5–5.9)	4.3 (4.2–4.5)	16.6 (12.9–21.0)	10.6 (7.7–14.2)	11.3 (8.3–15.0)	37.3 (28.6–47.8)	31.9 (23.9–41.7)	23.4 (16.7–32.0)
Deprivation									
Least deprived	3.2 (3.0–3.4)	1.9 (1.7–2.0)	2.0 (1.8–2.1)	6.4 (4.2–9.2)	2.7 (1.4–4.8)	3.4 (1.9–5.6)	18.9 (11.2–29.9)	11.5 (5.8–20.7)	14.7 (8.0–24.7)
Second least deprived	4.0 (3.8–4.3)	2.1 (2.0–2.3)	2.5 (2.3–2.7)	6.3 (4.2–9.1)	3.8 (2.2–6.2)	5.0 (3.1–7.5)	22.9 (14.8–33.8)	14.6 (8.4–23.8)	10.1 (5.0–18.0)
Middle deprived	4.3 (4.0–4.5)	2.7 (2.5–2.9)	2.6 (2.4–2.8)	7.4 (5.3–10.0)	2.5 (1.4–4.2)	4.1 (2.6–6.2)	16.8 (11.4–24.0)	10.7 (6.4–16.7)	11.8 (7.3–18.0)
Second most deprived	5.1 (4.9–5.3)	3.7 (3.5–3.9)	3.2 (3.0–3.4)	7.3 (5.4–9.6)	4.2 (2.8–6.0)	4.4 (2.9–6.2)	20.9 (15.7–27.3)	14.7 (10.4–20.2)	14.7 (10.4–20.2)
Most deprived	5.4 (5.2–5.6)	4.2 (4.0–4.4)	3.4 (3.2–3.6)	8.0 (6.2–10.2)	3.9 (2.7–5.5)	4.7 (3.4–6.4)	19.2 (15.2–23.8)	14.7 (11.2–18.8)	14.2 (10.8–18.3)

GP, general practitioner; ED, emergency department.

**Table 4:** Number of events, incidence per 1000 person years at risk (95% CI) and adjusted IRR (95% CI) for recording of self-harm events across settings^a^

	Non-court			Private court			Public court		
Variable	GP	ED	Hospital admissions	GP	ED	Hospital admissions	GP	ED	Hospital admissions
Year									
2011	Reference (*p* < 0.001)	Reference (*p* < 0.001)	Reference (*p* < 0.001)	Reference *p* = 0.589	Reference *p* = 0.116	Reference *p* = 0.659	Reference *p* = 0.503	Reference *p* = 0.017	Reference *p* = 0.039
2012	1.2 (1.1–1.5)	1.3 (1.1–1.6)	1.2 (1.0–1.4)	1.5 (0.5–4.6)	3.4 (0.4–25.4)	2.4 (0.4–15.8)	1.4 (0.7–2.8)	1.9 (0.6–5.7)	0.9 (0.3–2.5)
2013	1.4 (1.2–1.6)	1.5 (1.3–1.8)	1.5 (1.3–1.8)	2.0 (0.7–5.7)	4.6 (0.7–31.6)	3.1 (0.5–19.5)	1.5 (0.8–2.8)	1.5 (0.5–4.5)	1.6 (0.6–4.2)
2014	1.2 (1.1–1.4)	1.4 (1.2–1.6)	1.4 (1.2–1.6)	1.5 (0.5–4.5)	1.4 (0.2–10.5)	2.0 (0.3–12.7)	1.1 (0.6–2.2)	3.2 (1.3–8.2)	2.2 (0.9–5.7)
2015	1.3 (1.1–1.5)	1.8 (1.6–2.1)	1.6 (1.4–1.9)	2.0 (0.7–5.3)	5.1 (0.8–34.9)	3.2 (0.5–19.0)	1.4 (0.8–2.7)	3.6 (1.4–9.6)	1.6 (0.6–4.0)
2016	1.2 (1.0–1.3)	1.5 (1.4–1.7)	1.3 (1.1–1.5)	1.8 (0.7–4.9)	3.2 (0.5–21.8)	2.8 (0.5–16.3)	1.5 (0.8–2.7)	3.1 (1.2–8.2)	1.7 (0.7–4.1)
2017	1.3 (1.1–1.4)	1.5 (1.3–1.7)	1.4 (1.2–1.6)	1.5 (0.5–4.0)	3.6 (0.5–23.9)	2.3 (0.4–14.3)	1.5 (0.8–2.7)	2.8 (1.1–7.3)	2.2 (0.9–5.4)
2018	1.3 (1.1–1.5)	1.6 (1.4–1.9)	1.3 (1.1–1.5)	1.8 (0.7–4.8)	4.6 (0.7–30.5)	2.2 (0.4–13.1)	0.9 (0.4–2.0)	2.2 (0.9–5.6)	1.4 (0.5–3.9)
Gender									
Male	Reference (*p* < 0.001)	Reference (*p* < 0.001)	Reference (*p* < 0.001)	Reference (*p* < 0.001)	Reference (*p* < 0.001)	Reference (*p* < 0.001)	Reference (*p* < 0.001)	Reference (*p* < 0.001)	Reference (*p* < 0.001)
Female	4.0 (3.7–4.3)	2.2 (2.1–2.4)	4.8 (4.4–5.2)	2.9 (2.3–3.7)	2.4 (1.6–3.5)	5.0 (3.3–7.6)	4.0 (2.9–5.5)	2.5 (1.8–3.4)	4.4 (3.1–6.3)
Age group									
10–14 years	Reference (*p* < 0.001)	Reference (*p* < 0.001)	Reference (*p* < 0.001)	Reference (*p* < 0.001)	Reference (*p* < 0.001)	Reference (*p* < 0.001)	Reference (*p* < 0.001)	Reference (*p* < 0.001)	Reference (*p* < 0.001)
15–17 years	2.4 (2.2–2.6)	3.4 (3.2–3.7)	2.2 (2.0–2.3)	2.9 (2.2–3.8)	4.2 (2.8–6.1)	3.4 (2.4–4.9)	2.2 (1.6–2.9)	2.8 (2.1–3.7)	1.9 (1.4–2.6)
Deprivation									
Least deprived	Reference (*p* < 0.001)	Reference (*p* < 0.001)	Reference (*p* < 0.001)	Reference *p* = 0.340	Reference *p* = 0.572	Reference *p* = 0.723	Reference *p* = 0.788	Reference *p* = 0.788	Reference *p* = 0.302
Second least deprived	1.3 (1.1–1.4)	1.1 (1.0–1.3)	1.3 (1.1–1.5)	1.0 (0.6–1.7)	1.4 (0.7–2.8)	1.5 (0.8–2.7)	1.2 (0.7–1.9)	1.2 (0.6–2.5)	0.7 (0.4–1.2)
Middle deprived	1.3 (1.2–1.5)	1.4 (1.3–1.6)	1.3 (1.2–1.5)	1.2 (0.8–1.8)	0.9 (0.4–2.2)	1.2 (0.7–2.3)	0.9 (0.5–1.4)	0.9 (0.4–1.9)	0.8 (0.5–1.4)
Second most deprived	1.6 (1.4–1.8)	2.0 (1.8–2.2)	1.6 (1.4–1.8)	1.2 (0.8–1.8)	1.6 (0.8–3.0)	1.3 (0.8–2.3)	1.1 (0.7–1.7)	1.3 (0.7–2.4)	1.0 (0.6–1.7)
Most deprived	1.7 (1.5–1.9)	2.3 (2.1–2.5)	1.7 (1.6–1.9)	1.3 (0.9–2.0)	1.5 (0.8–2.8)	1.5 (0.8–2.6)	1.0 (0.6–1.5)	1.3 (0.7–2.3)	1.0 (0.6–1.6)

IRR, incident rate ratios; GP, general practitioner; ED, emergency department.

a. Adjusted for gender, age, group, deprivation and year.

Incidence of self-harm was higher in the most deprived than least deprived areas across all settings for the non-court group (non-court IRR most deprived GP 1.7 (1.5–1.9); emergency department 2.3 (1.6–2.9); hospital admissions 1.7 (1.6–1.9)). Incidence of self-harm was higher in more deprived areas in both the private and public court groups; however, this difference was not statistically significant.

For non-court children, incidence of self-harm increased across all settings over time (adjusted IRRs GP 1.3 (1.1–1.4); emergency department 1.6 (1.4–1.9); hospital admissions 1.3 (1.1–1.5)). Rates of self-harm increased in the private court group across all settings, but this did not reach statistical significance when adjusted for age and gender. For the public court group, emergency department attendances increased from 4.7 (1.0–13.8) cases per 1000 PYAR in 2011 up to 18.6 (12.4–26.9) in 2015 (IRR 3.6 (1.4–9.6)) with a decrease in incidence from 2016 onwards resulting in a non-significant increase overall (IRR 2.2 (0.9–5.6)). While rates of self-harm were higher for private and public court groups, it should be noted that small numbers were present when this was broken down by year and, as such, confidence intervals were wide.

### Time-to-event analysis

Over three quarters of the children involved in private (79.4% (78.1–80.7; 13 530/17 041)) and public (69.5% (67.3–71.7; *n* = 3838/5524)) court proceedings were under the age of 10 (median age 6 years) at time of initial court application date. Over half of the private court (53.1% (52.0–54.2); *n* = 9052/17 041)) and two thirds (68.5% (66.3–70.7; *n* = 3784/5524)) of the public court group were living in the two most deprived quintiles compared with 44.5% (44.1–44.9; *n* = 101 454/225 650) of the matched cohort and the time of court registration/match date.

### Self-harm outcomes

Out of the 17 041 private court-involved children 8872 had data available after their 10th birthday (to meet the criteria for identifying self-harm). Of these 2.6% (2.2–2.9; *n* = 227) had a self-harm outcome before the censor date. The average follow-up time to the first self-harm event before censorship for the private court group was 3.5 years. Out of the 5524 public court cases 2964 had data available after their 10th birthday of which, 5.5% (4.7–6.4; *n* = 162) had a self-harm outcome event by the study censor date. The average follow-up time to the first self-harm event before censorship for the public court group was 1.9 years. The majority of children involved in private law proceedings, with a self-harm event in the follow-up period, had no history of self-harm at baseline (96.0% (83.7–109.7), 218/227)), compared with around three-quarters (74.7% (62.0–89.2), 121/162)) of the public court group with no previous history of self-harm at baseline.

After adjusting for deprivation and previous history of self-harm, children involved in private law proceedings had over double the risk of self-harm than the age- and gender-matched non-court cohort (hazard ratio 2.0 (1.7–2.2)) following court involvement. Children who had been involved in public law proceedings had around three times the risk of self-harm than the non-court cohort (hazard ratio 3.2 (2.7–3.8); Table 5).

**Table 5:** Hazard ratios (95% CI) for self-harm following court registrations (case-control cohort)^a^

Variable	Unadjusted		Adjusted^b^	
	Private court	Public court	Private court	Public court
All	2.0 (1.8–2.3)^*^	5.1 (4.3–6.0)^*^	2.0 (1.7–2.2)^*^	3.2 (2.7–3.8)^*^
Males	2.2 (1.7–2.9)^*^	4.5 (3.2–6.2)^*^	2.2 (1.7–2.8)^*^	3.0 (2.1–4.3)^*^
Females	2.0 (1.7–2.3)^*^	5.4 (4.5–6.5)^*^	1.9 (1.6–2.2)^*^	3.4 (2.7–4.1)^*^
Least deprived	2.9 (2.0–4.3)^*^	11.8 (7.2–19.3)^*^	2.9 (2.0–4.2)^*^	4.5 (2.4–8.4)^*^
Most deprived	1.6 (1.2–2.0)^*^	3.8 (2.9–4.9)^*^	1.6 (1.2–2.0)^*^	3.1 (2.4–4.0)^*^
No history of self-harm	2.0 (1.8–2.3)^*^	4.0 (3.4–4.9)^*^	2.0 (1.7–2.3)^*^	3.7 (3.1–4.5)^*^
History of self-harm	1.5 (0.7–3.1)	1.7 (1.1–2.5)**	1.5 (0.7–3.0)	1.7 (1.2–2.6)**

a. Non-court as reference group.

b. Adjusted for deprivation and/or history of self-harm. No adjustment made for age and gender as the control group was matched on these characteristics.

* *p* < 0.001; **p < 0.05.

After adjusting for area-level deprivation and history of self-harm we found the risk of self-harm following both private and public court procedures were comparable for males and females. When adjusting for history of self-harm, hazard ratios were higher for those from the least deprived than the most deprived areas (adjusted hazard ratio private court least deprived 2.9 (95% 2.0–4.2), most deprived 1.6 (1.2–2.0); public court least deprived 4.5 (2.4–8.4), most deprived 3.1 (2.4–4.0)). When adjusting for deprivation, hazard ratios were higher for those with no history of self-harm than those with a history of self-harm (adjusted hazard ratio private court no history of self-harm 2.0 (1.7–2.3), history of self-harm 1.5 (0.7–3.0); public court no history of self-harm 3.7 (3.1–4.5), history of self-harm 1.7 (1.2–2.6)).

## Discussion

To our knowledge, this is the first study to quantify self-harm in children involved in private and public family court proceedings compared with the general population, utilising linked family justice (Cafcass Cymru) and healthcare data. Incidence of self-harm across all healthcare settings was higher for the court-involved children. Those involved in private courts had around twice the incidence of self-harm as the no-court cohort and those involved in public family court proceedings 4–5 times the rate (adjusted for age, gender and year). Incidence of self-harm was higher for females and older age groups. While, as expected, higher rates of self-harm were found in those from more deprived areas in the non-court group, this was not the case for the court-involved children. Adjusted IRRs showed an increase in self-harm over time for the non-court group across all healthcare settings. While rates of self-harm increased in both the private and public court groups this did not reach statistical significance.

Exploration of new incidences of self-harm as an outcome following court involvement compared with an age- and gender-matched cohort demonstrated that children involved in private law proceedings had around twice the risk of self-harm and those involved in public law proceedings had around three times the risk after adjusting for deprivation and history of self-harm. While risk of self-harm overall was higher for females than for males, when adjusting for deprivation and history of self-harm, the risk of new incidences of self-harm following court proceedings was comparable for both genders. Time-to-event analysis showed greater hazard ratios for those from less deprived areas compared with those from more deprived areas and, in those with no history of self-harm compared with those with a history of self-harm than their age- and gender-matched comparisons. This doesn’t reflect higher rates overall in less deprived areas/those with a history of self-harm but, rather, highlights an increase in the risk of self-harm compared with children with similar histories without court involvement. This likely reflects involvement with family justice proceedings, or the circumstances surrounding this, bringing rates of self-harm to a rate comparable with children with other pre-existing vulnerabilities.

The increased risk of self-harm in the private and public family court groups reflects previous work showing increased rates of depression and anxiety in this group^13^ and adds to evidence of the vulnerability of children involved in both private and public court proceedings as well as higher rates of self-harm in youth involved with criminal justice.^23^ Previous work with Cafcass England data has found a higher prevalence of children with autistic spectrum disorders and physical disabilities in both private and public court cases and higher rates of learning disabilities in public courts.^24^ This is alongside the greater needs of parents, including higher levels of mental health diagnoses, learning difficulties, language difficulties, substance misuse, health service use, domestic violence and self-harm.^3,25,26^ Children are more likely to have been exposed to a range of ACEs including childhood maltreatment and household dysfunction, parental divorce and poor family relationships. These are already-known risk factors for self-harm in young people.^10^ The increased rates of self-harm here are likely to be at least partly attributable to higher rates of these pre-existing risk factors. Contact with Cafcass represents an opportunity to identify individuals who need additional support.

In keeping with previous research, the most deprived communities were over-represented in the court groups.^3^ Deprivation is associated with self-harm at the population-level.^19^ Incidence of self-harm in the current study was higher in more deprived areas for the non-court group only. Within the court groups, the most deprived areas were heavily over-represented. Deprivation data were updated annually for each individual and lack of a clear pattern may be partly attributable to movement between deprivation quintiles, for example, following a care or child arrangement order. In adults, levels of deprivation may influence engagement with mental health services.^27^ This may affect contact with healthcare settings for self-harm that are initiated by parents/caregivers. Furthermore, across all deprivation quintiles rates of self-harm were elevated in the court compared with the non-court groups. This means that court-experienced children in both the most deprived and most affluent areas have higher rates of self-harm than their non-court counterparts.

### Strengths and limitations

This study utilises large population-level data including family law records linked to healthcare data across both primary and secondary care, making it possible to quantify long-term outcomes. We included a high number of matched children per case. Results are likely generalisable to the rest of the UK. This evidence can be used to target where in the system improvements can be made both on a policy and practice level and how changes can best be integrated across services. However, studies based on administrative data are limited by the scope and quality of available data, which are collected primarily for administrative rather than research purposes. Further research is needed to compare self-harm following parental separation for families who utilise family courts and those who do not.

This study was guided by advocates for children throughout, including heads of children’s services across Wales. Future work through the Family Justice Data Partnership (FJDP) will benefit from co-production with young people through National Centre for Suicide Prevention and Self-Harm Research lived-experience initiatives.

Instances where an individual has self-harmed but has not presented to services, or where this is not recorded, will not be captured here and results likely underestimate self-harm in this population. This is a common feature of all studies with routinely collected data. These rates are intended to reflect self-harm where the individual presents to healthcare settings rather than rates at the community level. The longitudinal nature of health records allows for exploration of self-harm for a number of years following court proceedings. Limitations of the Cafcass data have been previously described.^18^ We acknowledge the possibility of some selection bias which can occur if subgroups of individuals have different linkage rates. However, 87% of the Cafcass Cymru records were successfully matched in SAIL, enabling linkage to health records, and we report characteristics of the final sample with GP data. This study includes only children who had at least 12 months of GP data. As such, it is possible that children with poorer mental health were excluded due to residential mobility,^28^ with mobility more common in children involved in public law proceedings.^29^

### Implications

Self-harm is one of the strongest risk factors for future suicide^8^ and the increased risk of self-harm in children in contact with family justice highlights the need for them to be prioritised in suicide prevention strategies. This should not be limited to those with care experience. Around a third of private court proceedings in Cafcass Cymru data have a marker of welfare concerns, and parents involved in care/public court proceedings are also a vulnerable group.^3,25^ The presence of parental mental health problems or substance misuse may compromise an adult’s ability to engage with support services outside of court, emphasising the need for child-focused outreach and support.

While self-harm rates were highest in the public court group, the number of children and rates of self-harm in children involved in private court proceedings supports previous calls that, in addition to addressing the dispute between adults, support needs to be readily and independently available for children during and after proceedings.^3,25^ Support would likely have the greatest impact if it was proactively offered rather than being dependent on the child or adult contacting services for help. Children involved in private law proceedings access variable levels of support from school-based support to Child and Adolescent Mental Health Services. However, it is not known how many children need mental health support or how many access it.^15^ Outreach could be offered in conjunction with regulatory reporting on the number of children engaged with, and the number who go on to seek, help to maximise engagement. The average length of time to a self-harm event (presenting to services) found here is 2–3 years. Consideration should be given to the support and monitoring of outcomes over an extended period. Self-harm specific support could be integrated into existing initiatives with a clear pathway of care (as suggested for non-self-harm specific support for separating families)^30^ promoted for both parents and services to ensure individuals are equipped to signpost families.

Further analyses are warranted to explore self-harm in the context of outcomes of proceedings (e.g. care, foster or stay with family) and to capture variation in family circumstances (e.g. parental separation, divorce or situations in which parents have never lived together) which may be a target for future policy strategies. Further research should capture other adverse outcomes, including substance misuse, absences and exclusions from school and educational attainment. A better understanding of a full range of outcomes will contribute to a better understanding of the scale, breadth and depth of impacts, which the family courts must consider during proceedings and beyond. Children’s mental health needs are a significant factor in placement stability/instability.^31^ Better understanding of the circumstances and needs of young people involved in court proceedings, including ensuring their voices are heard in proceedings,^32^ will enable tailored policy and practice responses and more carefully targeted intervention and support, both during the court process and beyond.

Policy makers need to be attentive to the challenges parents face when bringing up children in impoverished circumstances. The impact of welfare reforms, and reductions in services, on the well-being of vulnerable families warrants further exploration and policies designed to address child poverty need to be integrated with more targeted safeguarding policies.

To our knowledge, this is the first study to quantify self-harm in children involved with private family courts and is the second study to compare outcomes in court-involved (both private and public) children with a general population comparison. Pro-active self-harm and mental health support should be available for children in contact with family law proceedings. This should be integrated across social care, health and school settings and account for the potentially complex needs of both children and adults/carers. Further work is needed to explore the impact of the outcomes of proceedings and a full range of adverse outcomes.

## Data Availability

The data used in this study are available in the SAIL Databank at Swansea University (Swansea, UK) via the Adolescent Mental Health Data Platform (ADP), but, as restrictions apply, they are not publicly available. All proposals to use SAIL data are subject to review by an independent Information Governance Review Panel (IGRP). Before any data can be accessed, approval must be given by the IGRP. The panel carefully considers each project to ensure proper and appropriate use of SAIL data. When access has been granted, it is gained through a privacy-protecting safe haven and remote access system referred to as the SAIL Gateway. SAIL have established an application process to be followed by anyone who would like to access data via SAIL, details of which can be found at https://saildatabank.com/data/apply-to-work-with-the-data/. Derived data supporting the findings of this study are available from the corresponding author (A.J.) on request at a.john@swansea.ac.uk.
